# Large intraperitoneal lipoleiomyoma in a pre-menopausal woman: a case report

**DOI:** 10.1186/s12957-021-02256-9

**Published:** 2021-05-08

**Authors:** Sara L. Schaefer, Amy L. Strong, Sheena Bahroloomi, Jichang Han, Michella K. Whisman, Jodi M. Wilkowski, Christina V. Angeles

**Affiliations:** 1 Department of Surgery, University of Michigan School of Medicine, 1500 E. Medical Center Drive, Ann Arbor, Michigan 48109-5932 USA; 2 Department of Microbiology and Immunology, The Geisel School of Medicine at Dartmouth, 1 Rope Ferry Road, Hanover, New Hampshire 03755 USA; 3 Department of Pathology, University of Michigan School of Medicine, 1500 E. Medical Center Drive, Ann Arbor, Michigan 48109-5932 USA

**Keywords:** Myolipoma of soft tissue, Lipoleiomyoma, Benign, Female, Pre-menopausal, Leiomyoma, Rare

## Abstract

**Background:**

Lipoleiomyoma is a rare, benign variant of the commonplace uterine leiomyoma. Unlike leiomyoma, these tumors are composed of smooth muscle cells admixed with mature adipose tissue. While rare, they are most frequently identified in the uterus, but even more infrequently have been described in extrauterine locations.

**Case presentation:**

We describe a case report of a 45-year-old woman with a history of in vitro fertilization pregnancy presenting 6 years later with abdominal distention and weight loss found to have a 30-cm intra-abdominal lipoleiomyoma. While cross-sectional imaging can narrow the differential diagnosis, histopathological analysis with stains positive for smooth muscle actin, desmin, and estrogen receptor, but negative for HMB-45 confirms the diagnosis of lipoleiomyoma. The large encapsulated tumor was resected en bloc. The patients post-operative course was uneventful and her symptoms resolved.

**Conclusions:**

Lipoleiomyoma should be considered on the differential diagnosis in a woman with a large intra-abdominal mass. While considered benign, resection should be considered if the mass is symptomatic, and the diagnosis is unclear or there is a concern for malignancy.

## Background

Lipoleiomyoma is a rare, benign variant of uterine leiomyoma [1–3]. Classically, lipoleiomyomas are located in the uterus, with few case reports describing the formation of this tumor in extrauterine locations such as the cervix, ovary, broad ligament, retroperitoneum, pre-peritoneum, and intra-abdominal [4–8]. The tumor is most commonly found in post-menopausal women in their sixth and seventh decades and is histologically characterized by mature adipose tissue admixed with smooth muscle. Clinical presentation varies depending on location, with intra-abdominal tumors causing symptoms secondary to mass effect. The current standard of treatment is resection if the tumor is symptomatic, the diagnosis is unclear, or there is a concern for malignancy.

### Case presentation

A 45-year-old woman presented to her gynecologist with 6 months of increasing abdominal cramping and marked distention despite a 35-pound intentional weight loss. She denied other systemic or gastrointestinal symptoms. Her medical history was notable for in vitro fertilization resulting in twin pregnancy 7 years prior. She was otherwise healthy with a body mass index of 27 and not taking any medications. Physical examination was remarkable for a non-tender, but firm and distended abdomen with palpable findings suspicious for a large mass. Lower extremity edema was not identified in either leg suggesting that the mass was not compressing the iliac veins and likely superior to the pelvis. All laboratory testing, including serum hCG, was within normal limits.

**Fig. 1:**
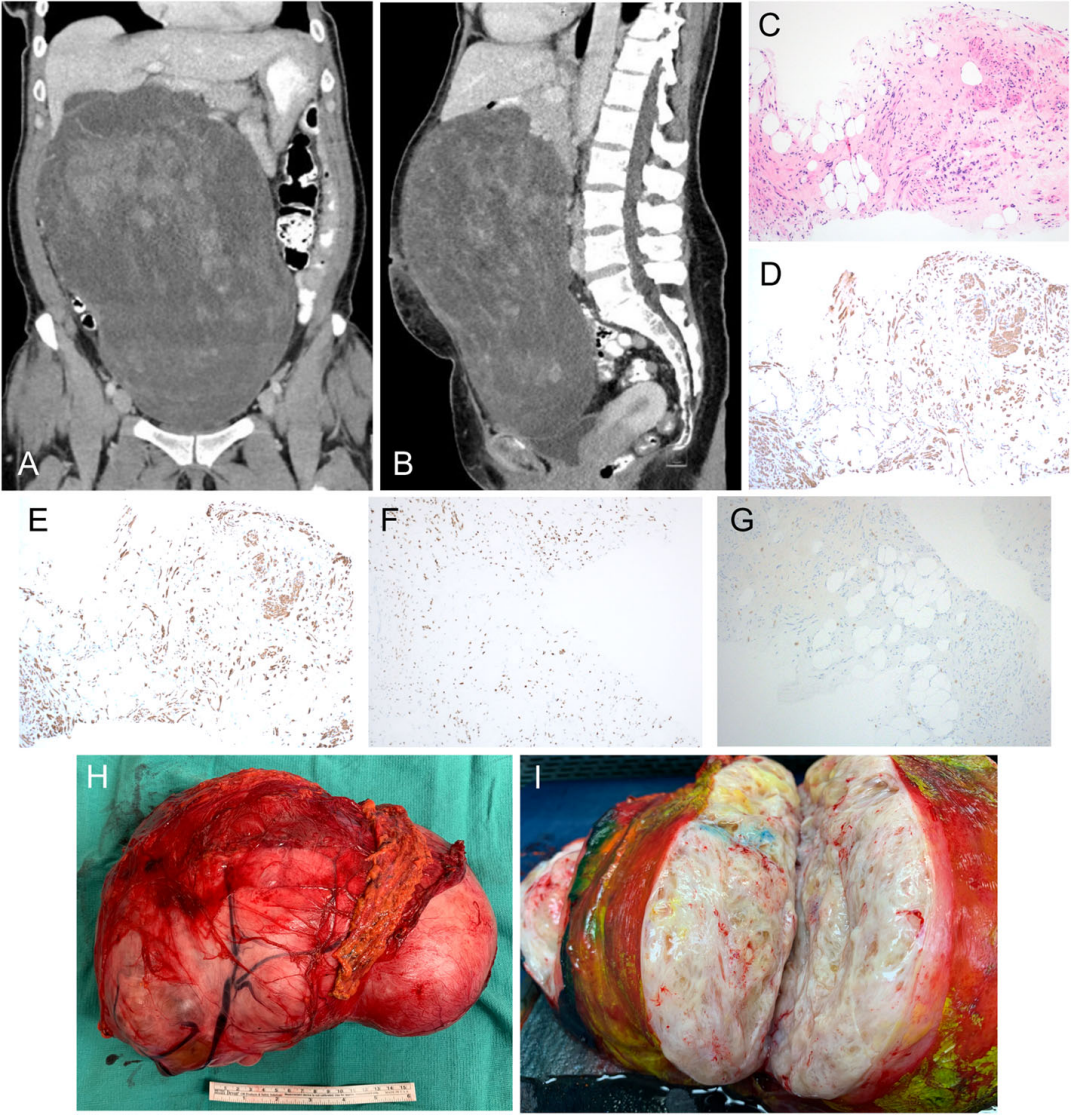
**a** Coronal CT demonstrates a heterogeneous hypodense intra-abdominal soft tissue mass, with adjacent transverse colon displaced into the pelvis. **b** Sagittal CT demonstrates the extent of the intra-abdominal mass spanning inferiorly to the pubic symphysis. **c** H&E stain of ultra-sound guided biopsies shows a hyalinized stroma containing an admixture of mature adipocytes and bland spindle cells with eosinophilic cytoplasm and fascicular arrangement, 10X. **d** Immunohistochemical stain shows strongly positive smooth muscle actin staining in the spindle cell component supportive of smooth muscle cell differentiation, 10X. **e** Immunohistochemical stain shows the spindle cell component expressing desmin, supportive of the smooth muscle cell, 10X. **f** Immunohistochemical staining shows spinal component expressing estrogen receptor, 10X. **g** Tumor cells are negative for HMB-45 immunoreactivity. The faint staining seen in the image is background mast cells appearing throughout the tumor. **h** Gross pathology specimen of resected mass with overlying omentum. **i** Cut section of a tumor with a distinct glistening appearance of the tumor

Computed tomography (CT) scan of the abdomen and pelvis revealed a large heterogeneous, hypodense mass measuring 27 cm x 20 cm x 13 cm with irregular, central solid-enhancing components (Fig. 1a, b). Based on cross-sectional imaging, the mass appeared distinct from the uterus, without associated ascites or lymphadenopathy. An ultrasound-guided core biopsy demonstrated an admixture of mature adipocytes and spindle cells. Immunohistochemical staining showed diffusely positive for smooth muscle actin, desmin, and estrogen receptor, but was negative for HMB-45 (Fig. 1c–g). Taken together with the imaging findings, the histopathological characteristics confirmed a diagnosis of lipoleiomyoma.

**Fig. 2:**
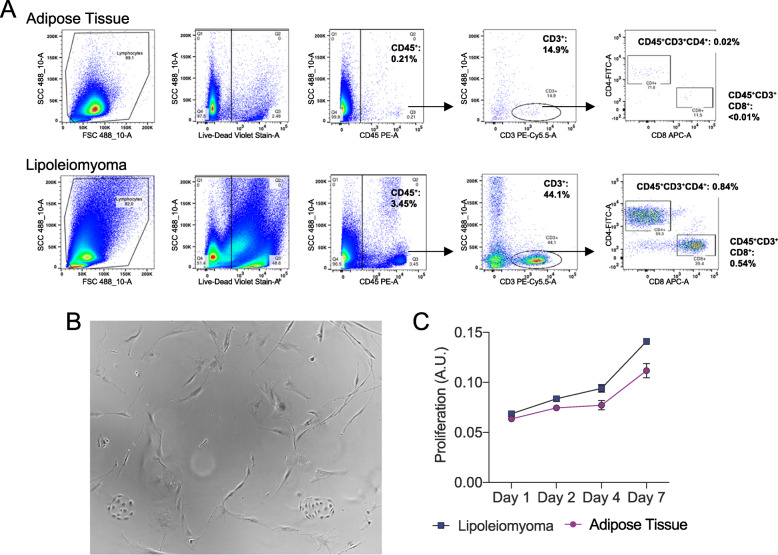
**a** Flow cytometry analysis demonstrates a small subpopulation of infiltrating T cells within the tumor which are increased compared to normal adipose tissue from the same patient. **b** Single-cell dissociation of the tumor demonstrates heterogenous population of cells composed of spindle cells, representative of fibroblast and smooth muscle cells, 10X. **c** MTT proliferation assay demonstrates tumor cells proliferate faster than the adipose tissue

Given the symptomatic nature of the mass, the patient underwent exploratory laparotomy to resect the tumor. Upon entering the peritoneum, the mass was immediately identified and encompassed most of the abdominal cavity. The tumor appeared to originate below the inferior border of the stomach with attachments to the mesentery and omentum and displaced the transverse colon into the pelvis. The tumor was well vascularized with feeding arteries from the mesentery and a large draining vessel to the superior mesenteric vein. Grossly, the tumor appeared well-circumscribed and was removed intact without disruption of the capsule (Fig. 1h). Cut section of the tumor showed a fibrofatty appearance (Fig. 1i). The final pathology confirmed a 30 cm x 20.5 cm x 12.5 cm lipoleiomyoma. Flow cytometric analysis of the tumor revealed a small population of T cell infiltrate (CD45+, CD3+), with a greater proportion of both CD8+ and CD4+ infiltrates compared to normal fat from the same patient (Fig. 2a). However, these populations are smaller than what has been shown in aggressive soft tissue sarcomas, which is consistent with the benign nature of lipoleiomyoma. Culturing of the dissociated tumor revealed a heterogenous population of cells with characteristics of fibroblast, smooth muscle cell, and endothelial cells (Fig. 2b). Lipoleiomyoma cells demonstrated increased proliferation compared to the adipose tissue, which is consistent with the rapid proliferation of the tumor compared to normal adipose tissue (Fig. 2c). Overall, the patient’s post-operative course was uncomplicated, and she was discharged on post-operation day 3. At her 3-week follow-up visit, she was feeling well, and her surgical incision was healing appropriately.

## Discussion

Lipoleiomyoma tumors are most often identified in the uterus as a variant of the commonplace leiomyoma. In rare cases, these benign tumors have been identified outside the uterus and described as extrauterine lipoleiomyoma. This rare entity has also been classified as myelolipoma of soft tissue. Despite variations in nomenclature, these tumors are described as histologically indistinct with smooth muscle blended with varying degrees of mature adipose tissue. To our knowledge, less than 15 cases of intraabdominal extrauterine lipoleiomyoma have been reported while the majority of these cases (40%) are retroperitoneal [9–11]. Here, we describe a case of a large intraperitoneal lipoleiomyoma in a pre-menopausal female. Differential diagnosis of a large fatty intra-abdominal mass includes benign cystic ovarian teratoma, lipoma, well-differentiated liposarcoma, extra-adrenal myelolipoma, lipoblastic lymphadenopathy, and angiomyolipoma. Imaging plays an important role in distinguishing lipoleiomyoma from other diagnoses, specifically liposarcoma or teratomas which require surgical excision. Distinguishing characteristics of lipoleiomyoma on computed tomography (CT) scans include a well-circumscribed, heterogenous mass with low attenuation fatty components mixed with non-fat tissue density. CT is typically used; however, magnetic resonance imaging (MRI) can be used if CT is contraindicated or in order to better delineate the involvement of nerves or vessels if the tumor is abutting these structures. In our patient, given the CT scan and biopsy findings, MRI was not deemed necessary in the pre-operative work-up. However, imaging is limited in distinguishing between benign and malignant lipomatous tumors, and therefore, an image-guided core needle biopsy is recommended. Histopathological characteristics solidify the diagnosis as these tumors are classically desmin, SMA, S-100, and ER-positive, but HMB-45-negative. Taken together, imaging and immunohistopathological characteristics confirm the diagnosis of lipoleiomyoma.

The etiology of the lipoleiomyoma is unknown; however, several hypotheses have been proposed. Most widely accepted is lipomatous metaplasia of muscle cells, suggesting perhaps lipoleiomyoma lies on a continuum with leiomyoma [1, 8, 11]. Other theories include misplacement of adipose cells in the embryological period, perivascular extension of peritoneal or retroperitoneal fat, traumatic implantation, and infiltration of perivascular adipose cells around the blood vessels [1, 8, 11]. Previous case reports have attributed the extrauterine lipoleiomyoma to seeding after gynecological surgery, exogenous hormone influence, or metabolic disturbances with insulin-like growth factor abnormalities. While our patient has had no previous surgery or other co-morbidities, her history is notable for in vitro pregnancy. It is unclear whether this acted as a hormonal influence on her tumor, but it is interesting that these tumors are generally estrogen receptor-positive [1, 12, 13]. The tumor presented in the current case report similarly demonstrated high estrogen receptor expression, which would suggest that hormonal influences may play a role in tumor development or progression. These findings are consistent with other studies which have demonstrated these tumors express estrogen receptors and may be responsive to hormonal stimuli [1, 5, 14]. Finally, although most lipoleiomyoma has been described in older, post-menopausal women, in contrast, our patient is younger and pre-menopausal. This may also support the hypothesis that hormonal factors play a role in the pathogenesis or growth of these rare tumors. Further studies are necessary to clarify the possible role of hormonal stimulation and help elucidate the pathogenesis of these rare tumors.

While the number of cases of lipoleiomyoma remains limited, the tumor is generally considered benign, without the risk of local recurrence or distant metastasis after resection. In contrast, an isolated case report has demonstrated leiomyosarcoma arising from an uterine lipoleiomyoma, suggesting malignant transformation may be possible [15]. Subsequent case studies have not demonstrated malignant transformation, local recurrence, or distant metastasis. Our ex vivo and in vitro analysis of the tumor also supported a benign, but highly proliferative phenotype, leading to a large, non-invasive, and non-metastatic extrauterine tumor. Our flow analysis demonstrated minimal infiltrating T cells, which contrasts with aggressive liposarcomas, and additional analysis demonstrated that the isolated tumor cells had increased proliferation compared to normal adipose tissue.

The management of intraabdominal lipoleiomyoma is typically surgical resection. Unfortunately, most patients present with very large tumors given that these tumors push but not invade surrounding structures, and patients do not seek medical consultation until they are symptomatic. This is also typical of patients with retroperitoneal sarcomas which are known to have a median size of 20cm at presentation [16]. Patients who have lipoleiomyomas that are symptomatic, or with intraabdominal tumors with unclear diagnosis in which malignancy cannot be excluded, should be recommended for surgical resection as long as they have controlled co-morbidities and acceptable surgical risk [17]. Our patient demonstrated mass effect given the size of her tumor and her symptoms resolved following resection. In asymptomatic cases, it would theoretically be possible to monitor these tumors with surveillance imaging if lipoleiomyoma was confirmed by biopsy given their generally accepted benign nature. However, given the rarity of lipoleiomyoma, there are not sufficient case numbers to definitively exclude the possibility of a more aggressive phenotype with malignant transformation, so monitoring is recommended if not resected. If surveillance imaging demonstrated an interval growth in the tumor, proceeding to the operating room before a significant increase in size could reduce the morbidity of surgery. For small tumors, laparoscopic removal could be considered.

## Conclusions

Together, our case report suggests that although rare, lipoleiomyoma should be considered in the differential for intraabdominal masses and resection effectively treats the tumor. Additionally, our case is aligned with previous reports which suggest that a hyperestrogenic state may play a role in the pathogenesis of lipoleiomyoma; however, further study is needed to better define the origin of these rare tumors.

## Data Availability

Not applicable.

## Abbreviations

CT: Computed tomography
cm: Centimeter
H&E: Hematoxylin and eosin
MTT: (3-(4,5-Dimethylthiazol-2-yl)-2,5-diphenyltetrazolium bromide)
ER: Estrogen receptor
SMA: Smooth muscle actin
HMB-45: Human melanoma black-45
